# Optimizing antibiotic prescribing for acutely ill children in primary care (ERNIE2 study protocol, part B): a cluster randomized, factorial controlled trial evaluating the effect of a point-of-care C-reactive protein test and a brief intervention combined with written safety net advice

**DOI:** 10.1186/1471-2431-14-246

**Published:** 2014-10-02

**Authors:** Marieke B Lemiengre, Jan Y Verbakel, Tine De Burghgraeve, Bert Aertgeerts, Frans De Baets, Frank Buntinx, An De Sutter, on behalf of the ERNIE 2 collaboration

**Affiliations:** Department of Family Practice and Primary Health Care, Ghent University, De Pintelaan 185 6 K3, Ghent, 9000 Belgium; Department of General Practice, KU Leuven, Kapucijnenvoer 33, Leuven, 3000 Belgium; Department of Pediatric Pulmonology, Infection and Immune Deficiencies, Ghent University Hospital, De Pintelaan 185 K12D, Ghent, 9000 Belgium; Research Institute Caphri, Maastricht University, PB 313, Nl 6200 MD Maastricht, The Netherlands

**Keywords:** Child, Infant, Acute disease, Anti-bacterial agents/economics/, Anti-bacterial agents/therapeutic use, C-reactive protein/analysis, Cluster analysis, Communication, Parent satisfaction, Physician's practice patterns, Point-of-care systems

## Abstract

**Background:**

Despite huge public campaigns, there is still overconsumption of antibiotics in children with self-limiting diseases. Possible explanations may be the physicians’ and parents’ uncertainty about the gravity of the disease and inadequate communication between physicians and parents leading to lack of reassurance for the parents. In this paper we describe the design and methods of a trial aiming to rationalize antibiotic prescribing by decreasing this uncertainty and parental anxiety.

**Methods/Design:**

Acutely ill children without suspected serious disease consulting their family physician will be consecutively included in a four-armed cluster randomized factorial controlled trial. The intervention will consist a Point-of-Care C-reactive protein test and/or a brief intervention with safety net advice. The control group will receive usual care. We intend to include 2560 patients in 88 family practices. Patients will be followed up until cure. The primary outcome measure is the immediate antibiotic prescribing rate. Secondary outcomes are: comparison between groups of speed of clinical recovery, parental concern, parental perception of the quality of the communication, parental satisfaction, use of medication, use of diagnostic tests and medical services during the illness episode, and cost-effectiveness of the interventions. Besides this, we will observationally analyse data of the children included in the large ERNIE2-trial, but excluded in the cluster randomized trial, namely children suspected of serious disease presenting in primary care and children who initially present at the out-patient paediatric clinic or emergency department. We will search for predictors of antibiotic prescribing, speed of clinical recovery, parental concern, parental perception of communication, parental satisfaction, use of medication, diagnostic tests and medical services.

**Discussion:**

This is a unique multifaceted intervention, in that it targets both physicians and parents by aiming specifically at their uncertainty and concerns during the consultation. Both interventions are easy to implement without special training. When proven effective, they could offer a feasible way to decrease inappropriate antibiotic prescribing for children in family practice and thus avoid emergence of bacterial resistance, side effects and unnecessary healthcare costs. Moreover, the observational part of the study will increase our insight in the course, management and parent’s concern of acute illness in children.

**Trial registration:**

ClinicalTrials.gov Identifier: NCT02024282.

## Background

Acute illness is a common reason for encounter of children in family practice. The large majority of these children suffer from non-serious, self-limiting conditions. Less than 1% of acutely ill children visiting their family physician (FP) will have a serious illness [1]. Yet, many of these children will be treated with antibiotics; a Belgian continuous and integrated computerized morbidity registration network (Intego) [2] shows that about 45% of all children with an respiratory tract infection visiting their family physician receive an antibiotic prescription. This is still far too many and may lead to unnecessary adverse effects and costs, and to the emergence of antibiotic resistance. This overprescribing could partly be a reflection of the clinician’s diagnostic uncertainty and fear to deny antibiotics to a child with a serious bacterial infection. Another explanation may be the difficulty family physicians experience in convincing parents who are expecting antibiotics, that it is unnecessary.

### Physicians’ diagnostic uncertainty

In clinical practice, physicians can never guarantee that a child will recover as expected. There is always a small, but real, chance of complications. This uncertainty is uncomfortable: they fear being too late when the condition of the child deteriorates. Prescribing antibiotics in hope of preventing a bad outcome is one way of dealing with this uncertainty [3]. Previous research showed that physicians prescribe antibiotics more frequently when it is difficult to distinguish viral form bacterial causes [4–6]. A test helping to make this distinction may decrease antibiotic prescribing.

Another way to cope with uncertainty is creating a safety net, enabling parents to contact the physician promptly when the condition gets worse [7].

### Misconceptions of worried parents

Research has shown that there are many misconceptions about fever and its treatment. Parents expect symptom relief for their ill child and see antibiotics as the most potent treatment for this. They consider the severity and the impact of the illness on their child as more important grounds to prescribe antibiotics than its cause. In this mindset, any illness will improve quicker with antibiotics. When the physician does not prescribe antibiotics, some parents feel that their concerns have not been taking seriously [8].

Most parents understand that taking antibiotics too often could give problems including resistance or immunity, but only one out of three parents is worried their child is getting too many antibiotics. A minority of parents finds that the physician unjustifiably did not prescribe antibiotics to their child, or conversely, that the physician prescribed antibiotics unnecessarily [9].

These misconceptions can lead to pressure on physicians during the consultation. Previous research showed that physicians prescribe antibiotics more frequently when they think that patients expect antibiotics [10–12] or feel pressured by the patient or parent [9, 10, 13, 14].

### Miscommunication between physicians and parents

Physicians fail to communicate to parents how they assess an ill child and why they sometimes prescribe symptomatic treatments and at other times antibiotics. The well-intented quote “it’s a virus”, meant to soothe parents, is often interpreted as “the physician is unsure”. This increases the anxiety and the need for a second opinion. The anxiety grows further when different physicians express divergent opinions [8, 15].

Parents on the other hand fail to communicate their real concerns. Often they are very worried: they feel responsible for the wellbeing of their child and are afraid it will die or be irreversibly damaged. They visit the physician because they feel out of control, want to share the responsibility for the child and regain control [16]. Yet, they often feel uncomfortable or lack the confidence to express their anxiety and ask the physician questions about the treatment [8]. Moreover, parents are often in a dilemma as to when to seek advice: when the physician advises to wait and see, they feel they consulted too early and bothered the physician unnecessarily, but on the other hand, when the physician says they should have come earlier, they feel guilty [8].

### Parents’ need for information

Research shows that parents search for specific and practical information about how to care for their ill child, how to evaluate the cause and the severity of the illness, and the need for professional advice. They also want information about the illness, the most appropriate treatment and how to prevent their child from falling ill again. Parents think that being better informed could reduce their fears [8].

In this trial, we intend to evaluate the effect of a Point-of-Care (POC) C-reactive protein (CRP) test and a brief intervention combined with written safety net advice on the antibiotic prescribing rate in acutely ill children not suspected of serious disease in primary care.

Our hypothesis is that an objective technical tool, improved communication and provision of clear information for parents will decrease both the physician’s and parent’s uncertainty, increase their mutual understanding and thus decrease the need for an antibiotic prescription.

This trial is part of the ERNIE2-trial, which also comprehends a diagnostic study, validating vital signs, a symptom decision tree, a POC CRP-test and oxygen saturation in seriously ill children in urgent-access care [17].

### Research questions and outcome measures

Does performing a POC CRP test and a brief intervention with safety net advice in acutely ill children not suspected of serious disease in primary care, either separately or combined, have an effect on:

#### Primary outcome measure

Immediate antibiotic prescribing rate

#### Secondary outcome measures

1. Clinical recovery
2. Parental concern
3. Parental perception of communication
4. Parental satisfaction
5. Use of medication, diagnostic tests and medical services (including re-consultation) during the illness episode
6. Cost-effectiveness of point-of-care CRP testing, brief intervention with safety net advice

We will also collect observational data of the children included in the large ERNIE2-trial, but excluded in the cluster randomized trial, i.e. (1) children suspected of serious disease presenting in primary care and (2) children who initially present at the out-patient paediatric clinic or emergency department. We will search this data for predictors of antibiotic prescribing, speed of clinical recovery, parental concern, parental perception of communication, parental satisfaction, use of medication, diagnostic tests and medical services (including re-consultation) during the illness episode.

### Ethical approval

The protocol of this study was approved by the Ethical Review Board of the University Hospitals/KU Leuven, under reference ML8601. For more details, we refer to Verbakel et al. [17].

## Methods

The methodology is described according to the Consort 2010 statement for the reporting of cluster randomized trials [18].

### Design

We intend to perform a cluster randomized, factorial controlled trial in acutely ill children not suspected of serious disease presenting to a family physician (primary care). We will use a 2 × 2 factorial design to assess the effect of each intervention and to explore the effect of the interventions combined. There are four allocation groups, consisting of family physicians (1) using a POC CRP test, (2) applying a brief intervention with safety net advice, (3) using POC CRP test plus applying a brief intervention with safety net advice and (4) usual care (Figure one, Verbakel et al. [17]).

Besides this, we will also perform an observational study in children suspected of serious disease presenting in primary care and children who initially present at the out-patient paediatric clinic or emergency department.

### Participants

#### Clusters

The clustered trial includes family practices in Flanders (Belgium).

There is only one eligibility criterion: being able to recruit children consecutively during the inclusion period.

#### Patients

Children aged 1 month to 16 years, presenting to a FP (primary care) with an acute illness episode of maximum 5 days and scoring negative on a 5-stage decision tree (in order to exclude children with a potentially serious illness) will be included consecutively in the cluster randomized trial.

For more details about the decision tree and exclusion criteria, we refer to Verbakel et al. [17].

Children excluded in the cluster randomized trial, but included in the large ERNIE2-trial, will be included in the observational study.

### Interventions

Children included in the cluster randomized trial will receive the following interventions depending on the group to which the practice is allocated: a POC CRP test (group 1), or a brief intervention with safety net advice (group 2), or both (group 3), or none (group 4).

#### (I) POC CRP test (finger prick)

As POC CRP test, we chose the Afinion CRP test (the Afinion AS100 Analyzer, Alere, USA). For the rationale of this choice and details on the test’s performance and practical applications, we refer to Verbakel et al. [17]. All physicians will be instructed in performing the POC CRP test.

#### (II) Brief intervention with safety net advice

##### Brief intervention

As brief intervention the FP will ask the following three questions to the parents, namely: “Are you concerned?”, “What exactly concerns you?” and “Why does this concern you?”. This intervention was extensively piloted in different training practices, to guarantee that these questions provide us with the intended information.

FPs will implement this intervention without additional training. To ensure the questions are actually asked, we will request the physicians to register the answers.

##### Safety net advice

We will provide a parent information leaflet as safety net advice. It offers information about how parents can give some comfort to their ill child, which signs are important to follow up on and when they should contact their physician. This leaflet was based on literature concerning the management of fever and alarm symptoms [19–25]. To spread uniform messages to the parents, we made sure the leaflet was in accordance as much as possible with the information provided by Child and Family, an agency of the Flemish government for the well-being of young children and their families.

To test clarity and readability we asked parents of various education levels visiting a health care centre to read the leaflet and mark statements that were difficult to understand. On the basis of their remarks, we adjusted the leaflet.

We will ask the physician to give advice by means of this leaflet and to explain when the child should be re-evaluated.

Only physicians of group 2 and 3 will be informed about the content of the brief intervention and safety net. In accordance with the cluster design, all FPs within the same practice will belong to the same allocation group. To avoid contamination we will explicitly ask the physicians included in group 2 and 3 not to discuss these interventions with their colleagues participating in the other groups.

### Outcomes

#### 1. Cluster randomized trial

##### Immediate antibiotic prescribing rate

Physicians will record on the registration form whether they prescribed an antibiotic (yes/no). Besides this, they will note the type, the dose, the reason for prescribing, whether it is a delayed prescription (yes/no) and whether they thought the parents wanted antibiotics (yes/no).

As a measure for tendency to prescribe antibiotics, all physicians will complete a validated questionnaire measuring their “defensive attitude score”, which expresses the physicians’ “risk-avoiding attitude”. Physicians with higher scores prefer the certain to the uncertain [26]. We also registered the physician’s prescribing profile provided by the National Institute for Health and Disability Insurance (RIZIV, Belgium). This profile contains the percentage of their patients who were prescribed an antibiotic during 2011 and is an objective proxy for their general antibiotic prescribing behaviour.

When comparing antibiotic prescribing rates between the intervention groups we will take into account the physicians defensive attitude and general antibiotic prescribing behaviour as possible confounders, besides other personal characteristics (e.g. sex, age, practice type,…).

##### Clinical recovery

Parents will record in a diary the degree of sickness on a 4-point scale (not ill – moderately ill – quite ill – very ill) and the presence of fever until their child has recovered. On the basis of this data, we will calculate the duration of the illness episode.

We will compare the speed of clinical recovery between children who got antibiotics and children who did not, while taking into account the preliminary diagnosis and clinical condition.

##### Parental concern

Parental concern will be approached in different ways: we will register its extent, cause and duration throughout the illness episode.

1. Extent of concern
  After the consultation, parents will score their degree of concern before and after the visit on a segmented numeric version of a visual analog scale (VAS) (0 – 10 integers). Besides this, parents will record daily their degree of concern on a 4-point scale (not worried – moderate worried – quite worried – very worried) in the diary.
2. Reason for concern
  As part of the brief intervention, physicians of group 2 and 3 will record the reason for concern on the registration form.
  Besides this, all parents will complete a questionnaire about their concerns. This survey was developed by our research team and is based on the literature about parental concern. In the questionnaire, we will ask the parents if and where they sought advice before contacting their physician, what concerns them (list of conceivable reasons + opportunity to add other reasons in free text) and how in their opinion the physician can improve reassurance for the parent and their child (list + opportunity to add free text).
  Furthermore, worried parents will note daily the reason for concern in the diary.
3. Duration of concern
  On the basis of the concern score in the diary, we will calculate the duration of the concern.
  We will describe reasons for concern and compare parental concern between (1) the intervention groups, (2) children who received antibiotics and children who did not, (3) children who were referred and children who were not, (4) different preliminary diagnoses.
  Besides this, we will investigate whether the size of the family has an influence on the extent of concern.

##### Parental perception of communication

The Parent’s Perception of Primary Care measure (P3C) is a practical, reliable, and valid measure of parents’ reports of paediatric primary care quality, consisting of six subscales (continuity, access, contextual knowledge, communication, comprehensiveness and coordination). In this trial, only the communication subscale (4 items) was used to assess parents’ perceptions of communication. This subscale demonstrated a good internal consistency (*α* = 0.83) [27]. After the consultation, parents will rate the items using a five-point scale ranging from “never” (0) to “always” (4). Scores will range from 0 to 16. Higher scores mean better parental perceptions of communication [27, 28]. This questionnaire was translated in Dutch forward and backward.

We will compare the perception of communication between the intervention groups.

##### Parental satisfaction

The Parental Medical Interview Satisfaction Scale (P-MISS) is a measure of parent satisfaction with the medical encounter, consisting of four subscales (physician communication with the parent, physician communication with the child, distress relief and adherence intend). The P-MISS is reliable and demonstrated a good construct validity [29]. In this trial, we omitted the items that assessed the physician communication with the child, because most of the children will be infants and toddlers. After the consultation, parents will rate the items using a five-point Likert scale ranging from “strongly disagree” (1) to “strongly agree” (5). Scores on this adjusted P-MISS (16 items) can range from 16 to 80. Lower scores indicate higher satisfaction with care. This adjusted P-MISS total score showed good internal consistency (α = 0.86) [27].

We will compare the parental satisfaction between the intervention groups.

##### Use of medication, diagnostic tests and medical services (including re-consultation) during the illness episode

Physicians will note the use of other diagnostic tests (blood test, chest radiography, urine test, other tests) and if there was a referral to a paediatrician or the emergency department of a hospital.

Parents will record daily the use of prescribed or over the counter (OCT) medication and whether they contacted their FP, their paediatrician, a FP on duty and/or the emergency department of a hospital.

We will compare the use of diagnostic tests and medical services between the intervention groups, taking into account the defensiveness of the physician’s prescribing attitude.

##### Cost-effectiveness of POC CRP testing, brief intervention with safety net advice

We will balance the costs of both interventions and the antibiotic prescriptions and investigate which intervention is most cost-effective.

#### 2. Observational study

A number of children will be included in the large ERNIE2-trial, but excluded in the cluster randomized trial. For this large group of children we will obtain all data collected on the registration forms (described above), data on the characteristics of their physicians and their practices. Besides this, for children included by the family physician, we will collect data from the booklets. In this data we will look for predictors of antibiotic prescribing, speed of clinical recovery, parental concern, parental perception of communication, parental satisfaction, use of medication, diagnostic tests and medical services (including re-consultation) during the illness episode using multivariate analysing techniques. We will compare these results to the results of the cluster randomized controlled trial.

### Sample size

In order to detect an absolute reduction in antibiotic prescribing of 15% (from 40% to 25%), with 80% power at a 5% significance level, an individually randomized study would need 600 patients (150 patients per group, 4 groups) (Table 1) [30].

**Table 1:** **Sample size calculation in cluster randomised trials**

*Sample size (two independent groups, dichotomous outcome variable)*[30]	n = ((p_C_. (100 – p_C_) + p_E_. (100 – p_E_))/δ_0_^2^). f(α,β) with p_C_ = the ‘success’ rate in the control group; p_E_ = the ‘success’ rate in the experimental group; δ_0_ = p_E_ – p_C_ = the relevant treatment effect to detect.
*Variance inflation factor*[31]	VIF = 1 + (m − 1)ρ (assuming the clusters are of a similar size) with m the average cluster size; 9ρ the estimated ICC
*Intraclass Correlation Coefficient*[31]	This coefficient is defined as the proportion of a measure’s total variance that is shared among members of defined clusters. The ICC takes a value of between 0 and 1.

For a cluster randomized design, standard sample size estimates require inflation by a factor to achieve the equivalent power of a patient randomized trial (Table 1). This inflation factor is commonly known as the ‘design effect’ or the ‘variance inflation factor’(VIF) [31]. To calculate the VIF, we need an accurate estimate of the intraclass correlation coefficient (ICC) (Table 1). If the treatment of patients who attend the same practice is very consistent and there is a large variation across different practices, the ICC will be relatively large. In this case it is difficult to attribute differences between the practices to the intervention that was randomly assigned per practice. Accounting for this kind of clustering is important to detect effects of interventions [32].

Because calculating the exact ICCs before starting the field study is not possible, we have to rely on reviews of other similar cluster randomized trials. Estimates of ICCs vary according to setting and type of outcome: estimates of ICCs for process variables are higher than those for patient outcomes, and estimates derived from secondary care are higher than those from primary care. ICCs for process variables in primary care were of the order of 0.05-0.15. Estimates for patient outcomes in primary care were generally lower than 0.05 [33].

We calculated the VIF for a favorable scenario (ICC = 0.05), and for an unfavorable scenario (ICC = 0.15) in the primary care setting. Our target mean cluster size was 21. Taking into account an ICC of 0.05, the number of patients needed in each arm would be 328. This number increases to 640 with an ICC of 0.15. In this scope, we will need to recruit 64 to 122 family practices.

### Randomization

We intend to use stratified and block randomization. Practices will be stratified according to practice type (solo, duo, group). In each stratum the practices will be divided in four blocks in order of their random number, generated by Excel’s Random Number feature. The first block of each stratum will be allocated to group 1, second block to group 2, third to group 3, and the fourth to group 4.

### Implementation

Recruitment of physicians and children is described in detail in Verbakel et al. [17].

### Data collection and follow-up

We will ask the FPs to perform a thorough history, physical examination and pulse oximetry in each child and register their findings on a case report form. For further details, we refer to Verbakel et al. [17]. At the end of the consultation, FPs will ask parents to complete a booklet (surveys, diary) at home until the child has recovered, to send a text message to the investigators on the day the child has recovered, and to post the booklet in a prepaid envelop to the investigators.

Small incentives and regular reminders for physicians and parents will be provided to stimulate inclusion rates and achieve complete data collection. More details are described in Verbakel et al. [17].

### Statistical analysis

The data will be stored and analyzed at two locations, KU Leuven and Ghent University, using Excel (Microsoft Corporation, Redmond, USA), Stata software (version 11.2; Stata Corp., College Station, TX, USA), SPSS (version 20; SPPS Inc., Chicago, Illinois, USA) and QSR NVivo version 10 (QSR International Pty Ltd, Melbourne, Australia).

Preliminary diagnoses and reasons for encounter will be classified using the International Classification of Primary Care (ICPC-2) [34]. Prescribed and OTC medication will be categorized using the Anatomical Therapeutic Chemical (ATC) Classification System [35].

Two investigators will independently code the reasons for concern and antibiotic prescribing following appropriate qualitative research methods.

The primary analysis will assess the effect of the two interventions on the primary and secondary outcome measures, using a three level logistic (dichotomous variables) or linear (continuous variables) regression model where appropriate, to account and correct for variation at the level of the practice (size, location, training practice), family physician (defensive attitude or general antibiotic prescribing behaviour, age, sex, years of experience, being in vocational training, graduated and/or vocational trainer) and patient (preliminary diagnosis, social background, size of the family, day of the week at inclusion). We will incorporate an interaction term to test for a synergistic (or antagonistic) relationship between the two interventions.

Besides this, we will observationally investigate the data of children included in the large ERNIE2-trial, but excluded in the cluster randomized trial. We will search predictors for antibiotic prescribing, reasons for concern and other secondary outcome measures as described above. We will compare these results to the results of the cluster randomized controlled trial.

## Discussion

This cluster randomized controlled trial will be the first to evaluate the effect of a POC CRP test and a simple communicative intervention with safety net advice on the antibiotic prescribing rate in acutely ill children not suspected of serious disease in primary care.

A Cochrane review investigating the effect of interventions to change the antibiotic prescribing behaviour found that multi-faceted interventions combining physician, patient and public education were the most successful in reducing antibiotic prescribing for inappropriate indications [36]. This intervention fulfils this condition by targeting both the physician’s and parental uncertainty: physicians are provided with a technical and communicative tool and parents receive clear information. Public education is not part of our intervention, but during this trial the annual national public health campaign to increase the awareness of the negative consequences of inappropriate antibiotic use (TV spots, folders, national antibiotic awareness day) will take place.

In the scope of the large ERNIE2-trial, we identified CRP as the most probable candidate to detect serious infections in febrile children in ambulatory settings and reduce irrational antibiotic prescribing. The Afinion AS100 Analyzer (Alere, USA) is a very user-friendly device, especially because a small drop of blood is enough to perform the test, making it perfect to use in children, and only two simple operations are needed to get the result (aspirating blood in the capillary, putting the cassette in the machine). Since currently, in children, there are no reliable cut-offs for CRP allowing to discriminate viral from bacterial causes, nor serious from non-serious illnesses, physicians did not receive any guidance on the interpretation of the CRP results, nor on the management. Finding reliable cut-offs is one of the goals of the ERNIE2-trial (part A) [17].

We are opting for a very simple communicative intervention to find out the worries of the parents by asking three short questions and handing out an information leaflet with alarm symptoms as safety net. We believe that a better knowledge of the ideas, concerns and expectations of the parents will help the physician to explain his preliminary diagnosis and treatment choices to the parents and draw attention to the alarm signs they should follow up on. We will ask the physicians to note the answers of the communicative intervention, so that we can ensure the questions were asked. We believe that this will help the physician to reassure parents, without an antibiotic prescription. Moreover, this trial will give us insight into parental reasons for concern. The existing trials investigating the worries of parents are rather old. New available sources of information (e.g. the internet) could have changed the nature of their concerns and information needs.

As far as we know, this combined intervention has never been executed before. We chose a simple intervention that can be implemented without training. We decided to make our leaflet corresponding to the information provided by Child and Family, which is an influential organization in Flemish health care. In this way we hoped to avoid that differences between information sources would lead to confusion and more uncertainty in parents.

We aim to include 2560 patients (following the unfavorable scenario), which should give the study enough power to yield clear significant results. The broad inclusion criterion will make the results applicable for a large group of acutely ill children attending in primary care. We believe that it will be possible to recruit such large number of children as acute illness in children is a very common reason for primary care encounters.

Randomization at practice level was chosen to avoid contamination of our communicative intervention. We will explicitly ask the physicians included in group 2 and 3 not to discuss this interventions with their colleagues participating in the other groups.

Recruitment rates across the four groups will be monitored closely. Practices recruiting poorly will be replaced. A multi-level analysis will be performed to take into account confounders, including defensive attitude or general antibiotic prescribing behaviour and preliminary diagnosis, which can influence the results. Results will be reported according to the Consort 2010 statement for reporting of cluster randomized trials [18].

POC CRP testing has previously been shown to reduce antibiotic prescribing in upper respiratory tract infections [37]. A trial with a similar design, combining POC CRP testing and a communicative intervention reduced antibiotic prescribing in coughing adults [38]. In this trial, we will test a similar intervention in acutely ill children. Still, we made some other choices concerning the interventions. Because of the lack of reliable cut-offs for CRP-testing in acutely ill children, physicians did not receive any guidance on the interpretation of the CRP results. Secondly, we choose not to actively train the family physicians to elicit patients’ concerns about their illness because we believe that interventions with training are difficult to implement afterwards. Moreover, the sample size will be considerably larger to yield enough power to compare the 4 treatment arms separately.

As far we know, the effect of a combination of a simple communicative intervention (without training) and a leaflet on antibiotic prescribing was not investigated before. A clustered randomized controlled trial found that the use of a interactive booklet alone on respiratory tract infections (RTIs) in children could lead to important reductions in antibiotic prescribing and the intention to consult without reducing parental satisfaction with care [39]. This intervention is similar, but differs at several points. In the trial of Francis et al, physicians were actively trained online to use the booklet to facilitate the use of certain communication skills. As stated above, our physicians were not trained. Contrary to our leaflet, the booklet focuses on upper respiratory tract infections instead of the general management of ill children and alarm symptoms.

If these interventions decrease the antibiotic prescribing rate or have favorable effects on parental concern and satisfaction, we will perform a cost-effectiveness analysis to evaluate the consequences of the intervention on the health care budget.

If the balance for one or more interventions is advantageous on irrational antibiotic prescribing and consequently on antibiotic resistance, we will promote the interventions in daily care by implementing them in guidelines and apply for reimbursement of POC CRP testing within the national health insurer for reimbursement.

## Abbreviations

FP: Family physician
POC: Point-of-care
CRP: C-reactive protein
VAS: Visual analog scale
P3C: Parent’s perception of primary care measure
P-MISS: Parental medical interview satisfaction scale
OTC: Over the counter
VIF: Variance inflation factor
ICC: Intracluster correlation coefficient
ICPC-2: International classification of primary care
ATC: Anatomical therapeutic chemical
RTIs: Respiratory tract infections.
